# Bilateral Proximal Tibia Stress Fractures through Persistent Physes

**DOI:** 10.1155/2018/8181547

**Published:** 2018-12-06

**Authors:** John J. Carroll, Sean P. Kelly, James N. Foster, Derek A. Mathis, Joseph F. Alderete

**Affiliations:** ^1^Department of Orthopaedic Surgery, Brooke Army Medical Center, Ft. Sam Houston, TX 78234, USA; ^2^Department of Pathology, Brooke Army Medical Center, Ft. Sam Houston, TX 78234, USA

## Abstract

**Introduction:**

Fatigue fractures are stress fractures resulting from repetitive trauma in areas of stress concentration. Prior case reports and studies have described stress fractures through persistent physes about the olecranon and distal fibula, as evidenced by hyaline cartilage on histologic analysis. However, there have been no documented proximal tibia stress fractures through persistent physes.

**Case Presentation:**

A 29-year-old military male basic trainee with varus alignment about his knees suffered bilateral medial tibial plateau stress fractures several weeks into military basic training. He underwent radiographic and laboratory evaluation of his stress fractures and eventual operative fixation of his bilateral tibial plateau fractures. Intraoperative specimens obtained from the fracture sites distal to the articular surface demonstrated abnormal fibrous appearing tissue. Histology demonstrated the presence of hyaline cartilage.

**Discussion:**

A 29-year-old military male basic trainee had bilateral proximal tibia stress fractures through persistent physes confirmed with biopsies demonstrating hyaline cartilage. Our belief is that the patient's persistent physes placed him at a greater risk for stress fractures and these may benefit from fixation in soldiers and athletes.

## 1. Introduction

Fatigue fractures are stress fractures resulting from repetitive injury at areas of stress concentration. Stress injuries, or overuse injuries, are very common in military recruits with one study demonstrating a 31% incidence of overuse injuries in basic military trainees [1]. Stress fractures of the proximal tibia have been rarely described, and treatment regimens are typically successful with rest and weight bearing restrictions [2, 3]. Rarer still is the reporting of stress fractures through a persistent physis. Several case reports involving the olecranon and distal fibula physes detail biopsies confirming hyaline cartilage at the fracture sites, but none have been reported in the proximal tibia [4–8]. This case report is the first reporting to our knowledge of both a persistent proximal tibia physis and a stress fracture through it.

## 2. Case Presentation

**Figure 1:**
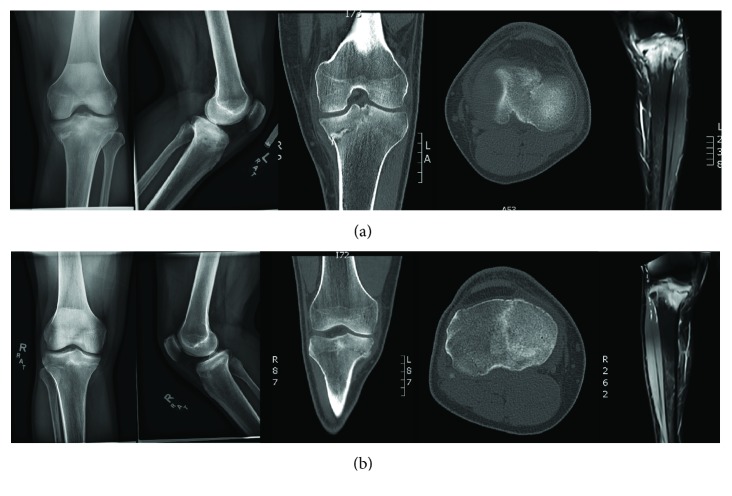
(a) Radiograhps, CT scan, and MRI of the left knee at the time of presentation demonstrate varus malalignment with medial sided proximal tibia stress fracture with extension to the articular surface and articular surface collapse. (b) Radiographs, CT scan, and MRI of the right knee demonstrate varus malalignment with medial sided tibia stress fracture without articular collapse.

**Figure 2:**
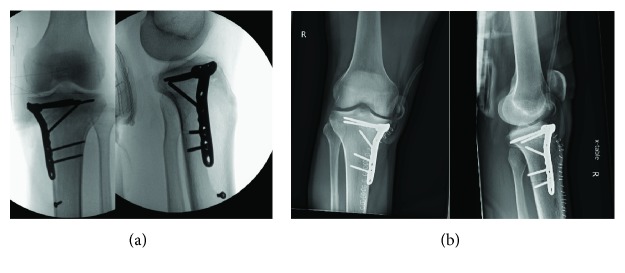
(a) Postoperative radiographs of left knee status post excision, grafting, and open reduction internal fixation left proximal tibia. (b) Postoperative radiographs of right knee status post excision, grafting, and open reduction internal fixation right proximal tibia.

**Figure 3:**
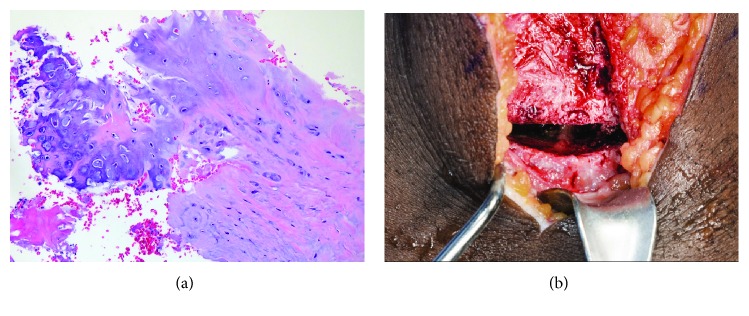
(a) Left proximal tibia specimen showing fragments of nonossifying hyaline cartilage with admixed fibroconnective tissue consistent with persistent physis (H&E; 200x). Histological examination of the left proximal tibia tissue specimen showed fracture callus comprising mixed chondroid/fibroconnective tissue undergoing ossification. Examination of the right proximal tibia tissue specimen obtained six weeks later demonstrated numerous admixed fragments of purple to purple-gray staining myxochondroid matrix suggestive of chondroid metaplasia, which is not an expected finding in this anatomic location distal to the articular surface. This presence of cartilage at this location is again consistent with a persistent physis. (b) An intraoperative photograph of right proximal tibia status post debridement of fibrous material at the site of prior physis.

A 29-year-old military male basic trainee (71 inches, 200 pounds, BMI 27.89) presented with a one-month history of atraumatic bilateral leg pain. He reported pain within the first week of initiating running at basic training. He was diagnosed with bilateral proximal tibia stress fractures at three weeks with instructions to stop impact activities. After failure of his symptoms to improve, he was referred to our facility. On presentation, he had tenderness about the medial aspect of each proximal tibia. Radiographs obtained at that time demonstrated bilateral proximal tibia stress fractures with varus alignment about each knee and articular collapse of the left tibial plateau. Metabolic labs were obtained and significant for a low vitamin D (15), but the remainders of labs including calcium, thyroid-stimulating hormone (TSH), and testosterone were unremarkable. MRI of the left knee demonstrated a medial tibial plateau fracture with two millimeters of articular depression and extension of the fracture to the tibial spine (Figure 1(a)). MRI of the right knee demonstrated a hypointense linear T2 signal surrounded by diffuse hyperintense signal suggesting a medial tibial plateau stress fracture with surrounding bone edema, without articular collapse or extension (Figure 1(b)). These fractures were at the level of the physis, and there was surrounding sclerosis on plain radiographs. Given the collapse of the articular surface in his left knee, the patient was indicated for open reduction internal fixation with allograft bone. The patient underwent the aforementioned procedure without complication, and the patient remained nonweight bearing to his bilateral lower extremities (Figure 2(a)). At the time of surgery, the bone at the fracture distal to the articular surface was softer and more friable in composition than expected for the stress fracture. It was easily debrided back to stable, healthy bone edges, and we were interested in analyzing its composition, so a biopsy was obtained and sent to the lab for analysis. Histology demonstrated nonossifying hyaline cartilage with admixed fibroconnective tissue consistent with a persistent physis (Figure 3(a)).

At the six-week follow-up visit after the procedure on his left knee, the patient was still having significant pain in his right lower extremity and, after extensive counseling, elected to undergo the same procedure on the contralateral limb in an effort to return to running and continue his military career (Figure 2(b)). We suspected that a similar pathology of a persistent physis was present in the right proximal tibia and that this was contributing to his slow healing progression. Similar intraoperative pathology specimens were again obtained at the fracture site distal to the articular surface (Figure 3(b)), and these demonstrated fragments of purple to purple-gray staining matrix with chondroid metaplasia. This presence of cartilage distal to the articular surface was consistent with a persistent physis.

At four-and-a-half months from the index procedure (three months from the staged procedure), the patient was ambulating without assisted devices and had painless range of motion from 0 to 120° in each knee with well-healed incisions, maintained hardware and alignment, but had not returned to impact activities. Due to his status as a basic trainee, he was released from active duty at six months' time postoperatively and was lost to follow-up.

## 3. Discussion

Our 29-year-old patient presented with bilateral proximal tibia stress fractures through persistent physes, which has not previously been reported in the literature. Typically, the proximal tibia physis closes at 18-19 years of age in males and 16-17 years of age in females [9]. The presence of hyaline cartilage in an adult patient outside of a joint surface has previously been described a persistent physis [10]. Biopsies from our patient demonstrated nonossifying hyaline cartilage with admixed fibroconnective and myxochondroid tissue. Our biopsy locations were intentionally distal to the articular surface to ensure no cross contamination from an intra-articular fracture.

With regard to nonphyseal stress fractures, the tibia is the most common site of stress fractures (49.1%), with the most common location of stress reaction being the posteromedial diaphyseal cortex [11]. However, medial tibial plateau stress fractures have been reported in the literature in runners and military personnel successfully treated with activity cessation, but none demonstrated articular collapse [2, 3]. These stress injuries often occur at the tibial metaphysis as well and are typically posterior and medial where weight bearing stress is greatest [3].

When evaluating stress fractures, factors that need to be considered include metabolic abnormalities and malalignment. To evaluate metabolic contributions to stress fractures, a laboratory workup should be completed. This should include a complete metabolic panel, including serum calcium, albumin, alkaline phosphatase, serum vitamin D levels, as well as calculated GFR, and relevant hormonal levels (thyroid and sexual hormones) [12]. As our patient's vitamin D was depressed, supplementation was instituted. Additionally, providers must consider malalignment and the additional stresses that this can expose the bone to. Our patient's varus alignment about the knees could have contributed to his development of stress fractures. The presence of stress fractures about the proximal tibia with malalignment has been described in osteoarthritic varus knees [13]. There are numerous causes of varus malalignment in pediatric and young adult patients to include physiologic bowing, Blount's disease, rickets, Osteogenesis imperfecta, and trauma [14]. Our patient did not have a previous diagnosis for his deformity, history of trauma, or clinical manifestations of syndromic disorders.

Prior studies have reported on the successful treatment of proximal olecranon stress fractures in throwers through persistent physes with resection, bone grafting, and internal fixation [5, 6, 15]. Sclerotic change about the physis on radiographs is a strong predictor of the need for operative management [16]. Our patient's stress fractures were at the level of the physis and had surrounding sclerosis. We felt that our patient's articular depression necessitated surgical correction on the left tibial plateau and, despite the tension mechanism of failure of the olecranon differing from the compression failure of the proximal tibia, this literature involving the proximal olecranon favored resection with bone grafting. The appearance of the bone at the fracture was quickly and easily debrided to healthy bone edges and suggested to us that something different than a standard stress fracture was present. Biopsy from the fracture site at the time of surgery confirmed a persistent physis. Given our patient's persistent symptoms on the contralateral extremity during follow-up and similar presenting imaging, we suspected a similar pathology in the right proximal tibia. Had this patient not been an active duty soldier in basic training and prolonged cessation of impact training was a viable option, we would have considered prolonged observation. Additional treatments such as ultrasound stimulation were considered, but this has taken up to seven months in the olecranon for ossification to occur [17]. Our intent was to return this patient to active duty within three to six months to give him a chance of completing basic training. After extensive discussion with the patient taking into account the suspected persistent physis in the right proximal tibia at the fracture, we decided to proceed to resection, bone grafting, and internal fixation. The patient did well from this postoperatively but was not able to return to running and was discharged from the military prior to completing basic training.

Stress fractures of the proximal tibia may occur through persistent physes. Things that suggest a persistent physis being present include a visualized physis on imaging, surrounding sclerosis on radiographs, and easily debrided material at the fracture site creating a cavitary lesion or void at the level of the suspected physis. Studies have demonstrated the successful treatment of fractures through persistent physes about the olecranon with resection, bone grafting, and internal fixation. Nonoperative management with protected weight bearing is reasonable when treating nondisplaced proximal tibia stress fractures through persistent physes due to the compressive rather than tension forces occurring in the proximal tibia. For patients with articular collapse or for those active patients who do not have the time for prolonged weight bearing restrictions, resection, bone grafting, and internal fixation may allow these patients to more quickly heal from their injury. Further research is needed to investigate this but is challenging given the rare nature of this injury.

## 4. Conclusion

We present a 29-year-old male basic trainee with bilateral proximal tibia stress fractures with intraoperative biopsies demonstrating the presence of hyaline cartilage and persistent physes treated by excision, bone grafting, and internal fixation. Persistent physes of the proximal tibia may be prone to stress fracture and subsequent collapse of the articular surface and may benefit from resection and fixation when recognized in active duty soldiers and athletes.
