# Corrigendum to “Bilateral Proximal Tibia Stress Fractures through Persistent Physes”

**DOI:** 10.1155/2019/2813130

**Published:** 2019-07-22

**Authors:** John J. Carroll, Sean P. Kelly, James N. Foster, Derek A. Mathis, Joseph F. Alderete

**Affiliations:** ^1^Department of Orthopaedic Surgery, Brooke Army Medical Center, Ft. Sam Houston, TX 78234, USA; ^2^Department of Pathology, Brooke Army Medical Center, Ft. Sam Houston, TX 78234, USA

In the article titled “Bilateral Proximal Tibia Stress Fractures through Persistent Physes” [1], there was an error in the authors' affiliation, where “San Antonio Military Medical Center” should be changed to “Brooke Army Medical Center.” The correct affiliations are shown above. In addition, a disclosure should be added as follows:

The view(s) expressed herein are those of the author(s) and do not reflect the official policy or position of Brooke Army Medical Center, the U.S. Army Medical Department, the U.S. Army Office of the Surgeon General, the Department of the Army, the Department of the Air Force, or the Department of Defense or the U.S. Government.
